# Salol

**Published:** 1890-08-09

**Authors:** 


### THERAPEUTICS.

### SALOL.

It is too much the practice of those who introduce new drugs to assert their entire freedom from the dangers or drawbacks incident to the use of those that they are intended to supersede. That unpleasant results have frequently followed the free use of salicylic acid as hitherto prepared from impure phenol is well known, and salol was declared to be absolutely safe. Being, however, in reality but a compound of salicylic acid, that is to say, a salicylic phenyl ether, and decomposed by the pancreatic secretion into its constituents, the baselessness of such assertions is self-evident. Dr. Edmund Falk, of Berlin, has collected a number of cases in which symptoms of phenol poisoning have been observed, although never severe; probably, as Sahli suggests, from the gradual liberation of the phenol and its possibly combining with the acids of the secretions into more or less inert compounds. These symptoms include profuse sweating, though not so extreme or exhausting as in the case of impure salicylic acid, rigors, rarely, but seen by Herrlich, buzzing in the ears first felt about two hours after administration, and often ceasing though the drug was continued, and partial deafness observed by Koster.

The disturbances of digestion comprise eructation of sour fluid and less often nausea and vomiting, with pain in the epigastrium. These are usually slight, but when large doses have been given for the purpose of reducing the temperature, and especially in the course of enteric fever, the vomiting may be persistent and exhausting. Herrlich observed in the case of a woman suffering from chronic articular rheumatism, who had taken 24 grams in three days, violent irrepressible vomiting prolonged over many days with complete anorexia, great prostration and strangury, the urine being highly charged with phenol.

The urine in this and other cases was of a dark green hue. Josefowitsch observed like symptoms in a man aged 40, who had taken 22½ grams in four days on account of acute articular rheumatism. He had also albuminuria and lumbar pains; while Kobert, by giving salol to dogs, induced albuminuria and nephritis. Herrlich has also observed some paradoxical thermal phenomena, as a rapid rise of temperature following rigors in enteric fever, and in one instance urticaria appearing soon after an enema of 2 grams which passed off in the course of a day, but returned on the administration of sodic salicylate.

